# *Ulva rigida* Valorization into Poly(3-hydroxybutyrate), Organic Acids and Functional Ingredients

**DOI:** 10.3390/md21100537

**Published:** 2023-10-14

**Authors:** Tânia Leandro, Marco Teles, Joana S. Gomes-Dias, Mafalda Marques, Cristina M. R. Rocha, M. Manuela R. da Fonseca, M. Teresa Cesário

**Affiliations:** 1 IBB—Institute for Bioengineering and Biosciences, Bioengineering Department, Instituto Superior Técnico, Av. Rovisco Pais, 1049-001 Lisboa, Portugal; tania.leandro@tecnico.ulisboa.pt (T.L.); marco.teles@tecnico.ulisboa.pt (M.T.); mafalda.marques@tecnico.ulisboa.pt (M.M.); manuela.fonseca@tecnico.ulisboa.pt (M.M.R.d.F.); 2 Associate Laboratory i4HB—Institute for Health and Bioeconomy at Instituto Superior Técnico, Universidade de Lisboa, 1649-004 Lisboa, Portugal; 3 CEB—Centre of Biological Engineering, Campus de Gualtar, University of Minho, 4710-057 Braga, Portugal; joana.dias@ceb.uminho.pt (J.S.G.-D.); cmrocha@ceb.uminho.pt (C.M.R.R.); 4 LABBELS—Associate Laboratory, 4710-057 Braga, Portugal

**Keywords:** *Halomonas elongata*, *Ulva rigida*, poly(3-hydroxybutyrate), gluconic acid, 2-oxoglutaric acid, functional ingredients

## Abstract

*Halomonas elongata* 1H9^T^ is a moderate halophilic strain able to produce poly(3-hydroxybutyrate) (P(3HB)), a biodegradable plastic, and gluconic acid, a valuable organic acid with wide industrial applications. In this work, the green alga *Ulva rigida* was used as platform to produce cultivation substrates for microbial conversion as well as functional ingredients, targeting its full valorization. The liquor obtained by autohydrolysis presented the highest concentration of oligosaccharides and protein, being an interesting feedstock to produce functional ingredients. The acid and/or enzymatic hydrolysis liquors are adequate as substrates for microbial processes. Shake flask assays with *H. elongata* revealed that the N-rich liquor produced after acidic treatment was the best suited for cell growth while the N-poor liquor produced by the enzymatic treatment of acid-pretreated algae residues produced the highest P(3HB) titers of 4.4 g/L. These hydrolysates were used in fed-batch cultivations as carbon and protein sources for the co-production of gluconic acid and polymer achieving titers of 123.2 g/L and 7.2 g/L, respectively. Besides gluconic acid, the Krebs cycle intermediate 2-oxoglutaric acid, also called alpha-ketoglutaric acid (KGA), was produced. Therefore, the co-production of P(3HB) and acids may be of considerable interest as an algal biorefinery valorization strategy.

## 1. Introduction

The pace of climate change is accelerating at an alarming rate, presenting our world with unparalleled challenges. As such, it is imperative to find solutions at a rapid rate. Strategies anchored in bioeconomy play an important role here. The bioeconomy concept is based on the use of biological resources to produce goods and services for a more sustainable future [1]. In this area, one important step is the search for biological sources which could be exploited as feedstock to produce biochemicals and biomaterials.

Macroalgae have great potential as a sustainable feedstock due to their high carbohydrate content (25–60% of dry weight). Additionally, marine macroalgae (seaweeds) present high growth rates with no utilization of arable land or freshwater. Moreover, they present easier fractioning than terrestrial plants due to lack of lignin and offer the possibility for recovery of additional value-added products within a biorefinery context [2].

Recently, our group described the ability of gluconic acid production by *Halomonas elongata* 1H9^T^, a moderate halotolerant bacterium isolated from a solar salt facility [3]. That study reported a process for the co-production of gluconic acid and poly-3-hydroxybutyrate (P(3HB)) from glucose [4]. Gluconic acid is a valuable organic acid with applications in diverse areas such as in the food, pharmaceutical, chemical and construction industries [5]. At present, the main processes for industrial production of gluconic acid are microbial cultivations using the fungus *Aspergillus niger* or by acetic acid bacteria, in particular, by *Gluconobacter oxidans* [6]. P(3HB) is the most common member of the polyhydroxyalkanoates family (PHAs). These are biodegradable polyesters accumulated intracellularly by microbial cells as carbon and energy storage. Due to their thermal and mechanical properties, PHAs have been regarded as potential substitutes for petroleum-derived plastics [7]. Currently, the production of PHAs is not economically competitive with that of petroleum-derived plastics due to high production costs, with feedstock representing 50% of the total PHA production cost [8,9]. Therefore, the co-production of P(3HB) and other commercially valuable products such as gluconic acid may well contribute to increase the economic competitiveness of this important bioplastic.

The study of halophilic and halotolerant PHA-producing strains such as the case of members of the *Halomonas* genus has gained particular interest. The ability to grow under high salt concentrations discouraging the growth of contaminants opens the possibility to design open microbial cultivations and thus further reduce operational costs.

Green seaweeds such as *Ulva* spp. are particularly interesting, as they have a protein content of around 9–14%, an amino acid composition similar to legumes and soy, and bioactive peptides with antiviral or anti-inflammatory activity [10]. Besides cellulose in the cell wall, the carbohydrate fraction includes ulvan and starch. Ulvan is a complex sulfated structural polysaccharide mainly composed of a repetitive sequence of rhamnose sulfate, glucuronic acid, iduronic acid and xylose [11,12]. Several potential bioactivities have been ascribed to ulvan, including antioxidant, antiviral, anticancer, anticoagulant or immunomodulating [11].

The goal of this work was to explore the feasibility of different integral valorization strategies for the carbohydrate-rich fractions of *Ulva rigida* in line with the bioeconomy concept for a more sustainable future. For this goal, the production of sugar-rich liquors from this seaweed was optimized in the first stage. In the second stage, the different liquors were evaluated as potential functional ingredients and as substrates for the production by *H. elongata* 1H9^T^ of gluconic acid and P(3HB) in batch conditions. In the third stage, the best-suited liquors which favored the co-production of both gluconic acid and P(3HB) in bioreactor fed-batch cultivations were selected [4] and applied.

## 2. Results and Discussion

### 2.1. Biomass Characterization

The composition of *Ulva rigida* biomass is presented in Table 1.

As can be observed, the main constituent of this biomass is ash, representing nearly a third of the material, followed by carbohydrates (corresponding to a quarter of the biomass), with smaller amounts of protein and lipids. This distribution is in accordance with the data reported in the literature [13]. When considering food applications, the high ash and mineral content of this biomass becomes even more promising: *Ulva rigida* is reported to have a Na/K ratio associated with blood pressure regulation, as well as high amounts of magnesium and iron, with a daily dose of 8 g of this seaweed contributing to more than 50% of the daily recommended dose of these micronutrients [14]. It was not possible to determine the iduronic acid content due to a lack of calibration standards. Since this compound is a constituent of ulvan, the total uronic acid content of the seaweed is expected to be even higher than reported.

### 2.2. Production of Hydrolysates from Ulva rigida

**Figure 1:**
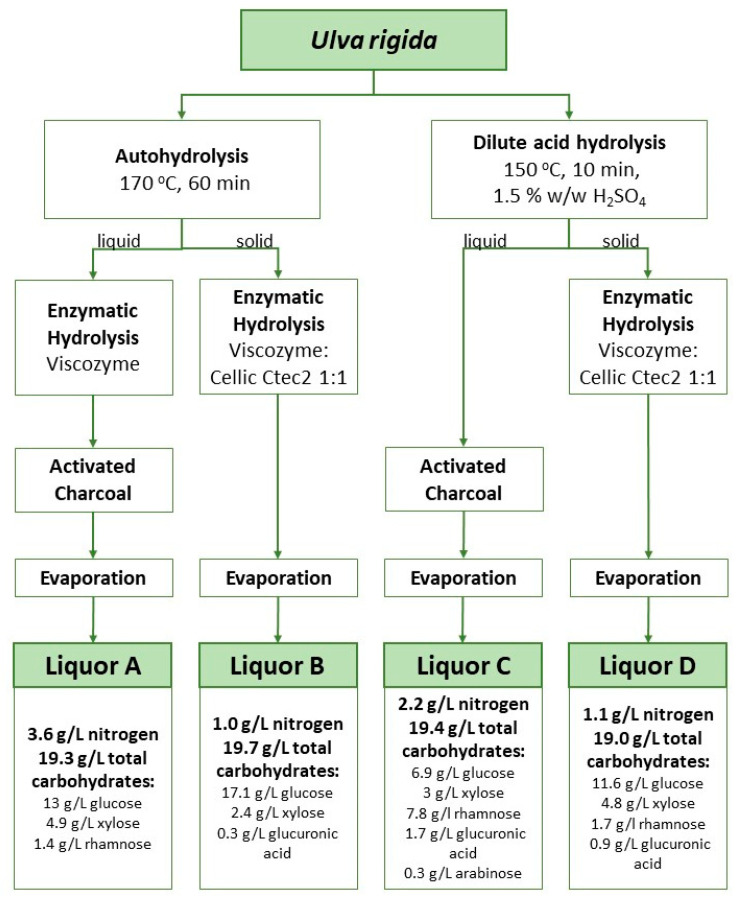
Schematic representation of liquor production from raw *Ulva rigida* using different combinations of hydrothermal, acidic and enzymatic hydrolysis, and composition of the liquors obtained thereof. Hydroxymethylfurfural (HMF) was kept between 0 and <0.1 g/L in all hydrolysates. Sugars were concentrated up to around 20 g/L. Total nitrogen determination using a nitrogen analyzer FP-528 DSP LECO (LECO, St. Joseph, MI, USA) according to the Dumas method.

The main structural polysaccharides in *Ulva rigida* are cellulose and ulvan, while starch is their storage counterpart. Ulvan is a complex polysaccharide typically containing sulfate ester and uronic acid groups as well as xylose, rhamnose and glucose residues. For their upgrade as substrates in biological processes, these polysaccharides need to undergo saccharification to simple sugars. Figure 1 shows the sugar composition of each liquor obtained after the application of four different combined treatments of chemical hydrolysis followed by enzymatic hydrolysis with a cellulolytic cocktail. The sugars released were glucose (from cellulose and starch), rhamnose and xylose (from ulvan) and glucuronic acid (also from ulvan). The ratio between these sugars was different depending on the applied treatment.

In the hydrothermal hydrolytic treatment (also referred to as autohydrolysis [15]), hydrolysis is started by hydronium ions resulting from water autoionization [16]. This type of treatment has energy costs, requires large quantities of water and a large fraction of the extracted sugars are recovered as oligomers [17,18]. Nevertheless, this is a chemical-free approach where water is used as the only solvent and the oligomers’ presence may be regarded as an opportunity to generate carbohydrate fractions with prebiotic features, besides the high soluble fiber content. In turn, drawbacks associated with acid hydrolysis involve not only an energy cost, but also costs associated with the disposal of wastes generated from the neutralization step. Moreover, the production of by-products is observed. These include 5-hydroxymethylfurfural (HMF) and furfural which result from the dehydration under acidic conditions of hexoses and pentoses, respectively. HMF has been described before with inhibitory action on bacterial cultivations [19]. The incorporation of an additional step of treatment with activated charcoal allowed the successful removal of most of this compound. As a result, HMF levels were kept below 0.1 g/L even after the last step of water evaporation aimed at the concentration of the extracted sugars (Figure 1). Enzymatic treatments present an attractive option for algae hydrolysis since they are less energy-demanding and do not generate wastes. However, their implementation at a large scale is challenging due to the cost of enzymes and extended reaction times. Additionally, because of the complex nature of macroalgal carbohydrates, enzymes with high specificity are not commercially available or involve very high prices. For instance, the polysaccharide ulvan in *Ulva rigida* requires a complex set of diverse specific enzymes for its degradation [20]. Therefore, enzymatic hydrolysis is often applied in conjunction with chemical treatments [21,22,23,24]. Hydrothermal or acidic treatments help not only to release sugars but also to loosen the structure of these complex carbohydrates and facilitate the posterior action of known enzyme cocktails. For instance, Cellic^®^ CTec2 can release monomeric sugars from polysaccharides such as glucans. Notwithstanding the drawbacks of chemical treatments, a combination of either hydrothermal or acidic steps with a subsequent enzymatic treatment is currently the best option to exploit macroalgal sugars as a carbon source in the cultivation of microbial strains which do not present the enzymatic machinery to metabolize the algal complex polysaccharides.

Liquors A and C had the highest nitrogen content with 3.6 and 2.2 g/L N, respectively. These values were expected because these liquors are derived from the liquid phase after chemical treatment and soluble proteins are extracted to the liquid phase during this step. On the other hand, Liquors B and D with 1.0 and 1.1 g/L N, respectively, were produced by enzymatic treatment of the solid phase, with the insoluble protein fraction present in the solids being released in this step.

### 2.3. Shake Flask Assays

**Figure 2:**
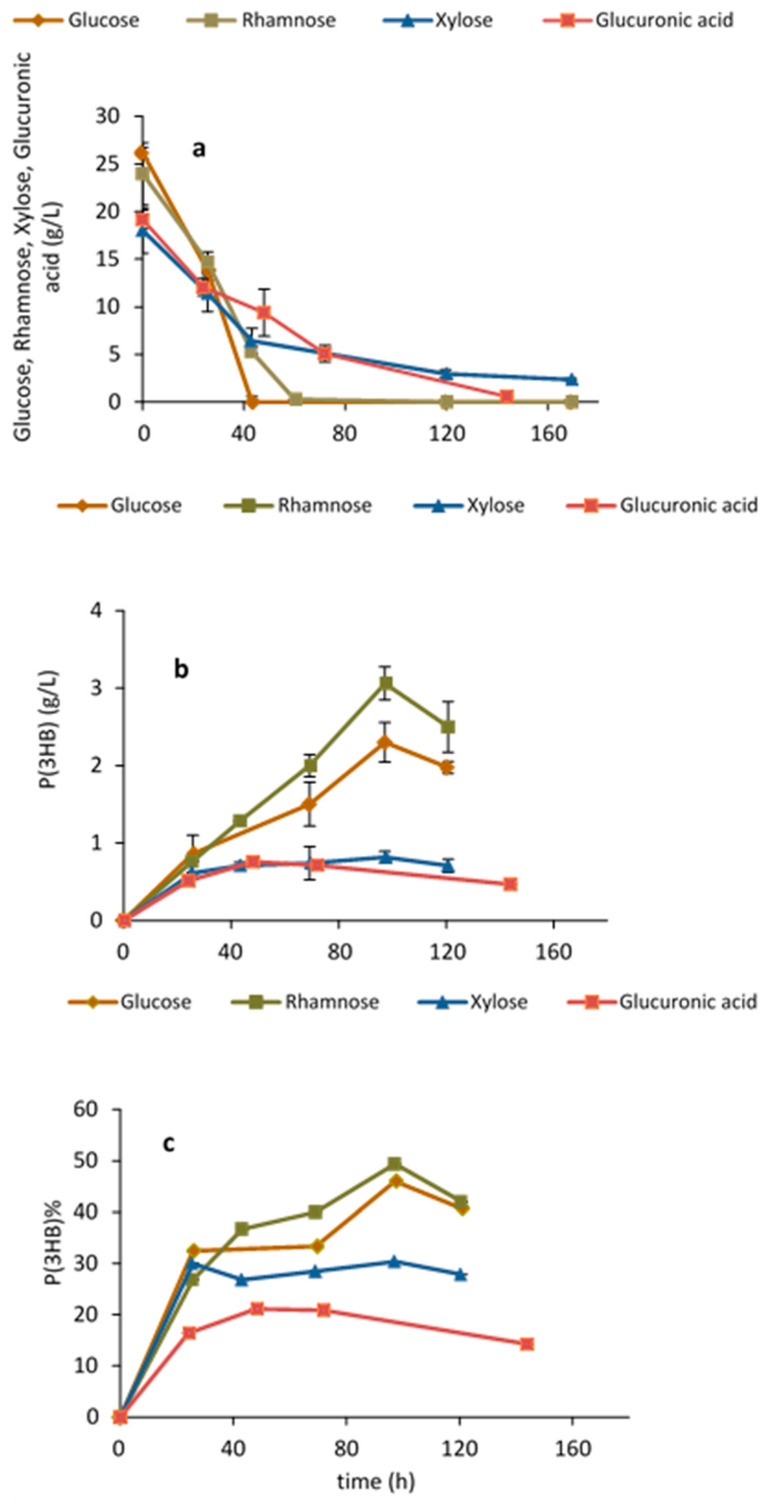
*Halomonas elongata* 1H9^T^ shake flask assays: (**a**) testing sugar utilization; (**b**,**c**) P(3HB) production and accumulation on glucose, xylose, rhamnose and glucuronic acid.

Growth and P(3HB) production by *H. elongata* 1H9^T^ on glucose has been previously demonstrated [25]. However, to the best of our knowledge, the capability of this strain for both growth and P(3HB) production on xylose, rhamnose and glucuronic acid, i.e., the sugars released after *Ulva* saccharification, has not yet been reported. Figure 2 shows growth and P(3HB) production on each of the different simple sugars and on the sugar acid as the sole carbon source. All sugars, in particular rhamnose, were able to support growth and production. P(3HB) accumulation on rhamnose was 49% *w*/*w* after 97 h, while on glucose an accumulation of 46% *w*/*w* was attained. The accumulation of P(3HB) on xylose and glucuronic acid was lower and attained values of 30% (*w*/*w*) and 21% (*w*/*w*), respectively. All the monomeric sugars released from *Ulva rigida* after hydrolysis of the carbohydrate fraction can thus be used by *H. elongata* for polymer production. This is the first report on the ability of *H. elongata* to grow and produce PHAs on rare sugars and sugar acids, namely, on rhamnose and glucuronic acid.

**Figure 3:**
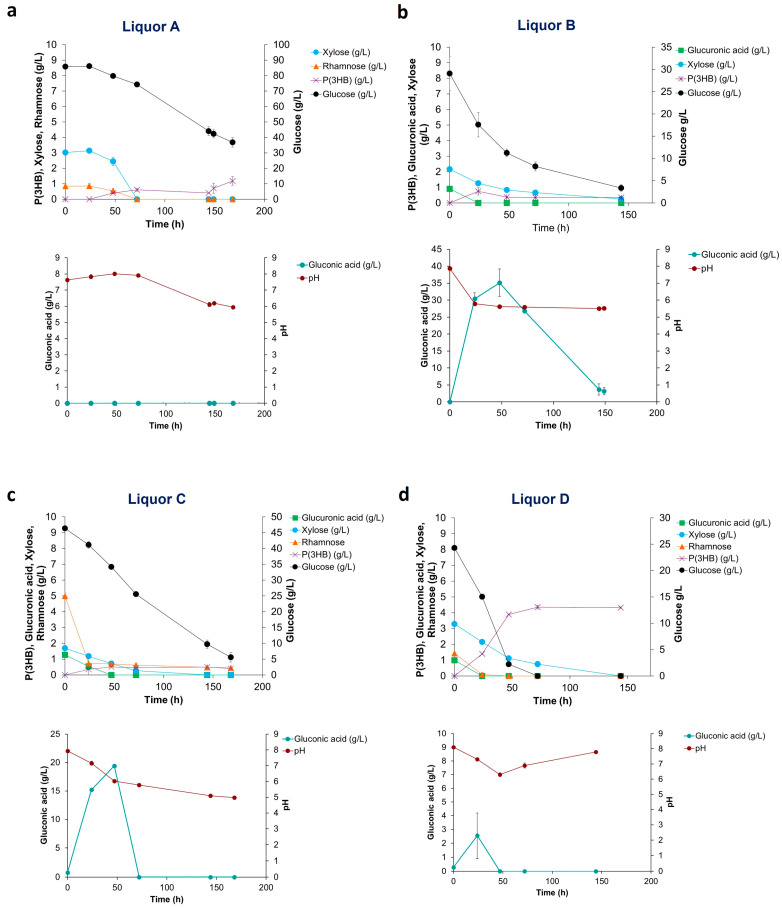
Sugar utilization, pH, gluconic acid and P(3HB) production of *H. elongata* 1H9^T^ growth on: (**a**) Liquor A; (**b**) Liquor B; (**c**) Liquor C; and (**d**) Liquor D produced from *Ulva rigida* carbohydrate saccharification.

The four *Ulva rigida* liquors (Figure 1) were tested as carbon and nitrogen source in shake flask assays. In these assays, no external nitrogen source was supplemented to the culture medium. A previous study by the authors has shown that *Halomonas elongata* 1H9^T^ is able to produce P(3HB) and gluconic acid under conditions of nitrogen limitation [4]. This is an advantage as both metabolites, intra- and extracellular, respectively, can be produced in one single cultivation, or alternatively, cultivation conditions should be selected to promote one or the other metabolite. Figure 1 shows that liquors A and C with 3.6 and 2.2 g/L N, respectively, have a higher nitrogen content, while liquors B and D with 1.0 and 1.1 g/L N exhibit a lower nitrogen content. From the literature, it is known that C:N ratios of 20–40 are needed to promote PHA production [26]. Since the sugar concentrations in the *Ulva rigida* liquors were low in relation to the total N content, commercial glucose was supplemented to each medium to reach a C:N ratio of 20. The direct quantification of cellular biomass during the assays was hindered by the presence of a sediment in all liquors. The concentration of sugars, the P(3HB) accumulation and the gluconic acid production were followed during the time course of the assays. The results of these assays can be observed in Figure 3.

Figure 3a shows that *H. elongata* when cultivated on Liquor A displayed an initial lag phase in sugar utilization. As the concentration of the inhibitor HMF in the hydrolysates is below 0.1 g/L, this initial delay could be related to growth inhibition caused by a high initial glucose concentration (90 g/L) (glucose was supplemented to reach a C: N ratio of 20). Following the first 48 h, sugar consumption started and the pH dropped slowly. At circa 150 h cultivation, an increased P(3HB) production rate was observed, which is probably related to low nitrogen titers attained after cell growth, i.e., N-limiting conditions had been finally reached. No gluconic acid was produced in this assay. When *H. elongata* was cultivated on Liquor B, sugars were rapidly used, leading to a pH decrease to values lower than pH 6 in the first 24 h (Figure 3b), affecting both growth and P(3HB) production. Maximum production of P(3HB) in this assay was 0.7 g/L reached at 24 h cultivation, while in the same time interval gluconic acid reached a concentration of 30 g/L. A maximum gluconic acid concentration of 35.1 g/L was attained at 47 h. It is observed that *H. elongata* preferably consumes the gluconic acid previously produced; however, this did not lead to P(3HB) accumulation. Thus, the conditions in this assay promoted gluconic acid instead of polymer production. When *H. elongata* was cultivated on Liquor C (Figure 3c), sugars were consumed, yet the pH dropped slower than what was observed with Liquor B. At 47 h, a maximum P(3HB) production of 0.5 g/L and a maximum gluconic acid production of 19.4 g/L were reached. As in Liquor A, the low P(3HB) production with liquor C could be explained by the high N content of the hydrolysate and because N-limiting conditions were not attained over the time course of the assay. High gluconic acid production with both Liquors B and C with residual co-production of P(3HB) was noteworthy, as this behavior had not been observed before. All sugars were consumed during the cultivation of *H. elongata* on Liquor D, yet pH values were always above 6.0 (Figure 3d). With this liquor, a maximum production of P(3HB) of 4.4 g/L at 72 h cultivation was observed, while a low gluconic acid concentration of 2.5 g/L was attained. The high P(3HB) production using Liquor D can be ascribed to a stable and neutral pH together with N-limiting conditions being reached. The stable pH value derives from the low gluconic acid production in this assay, which is also the reason for the higher polymer concentrations attained as sugars are channeled to polymer production. Liquors B and D had a similar composition (Figure 1); however, liquor B did not promote the production of P(3HB) due to the fast decrease in pH. In a cultivation system with pH control, the results with these hydrolysates might have been similar. The overall balance of the pH in each assay was a result of its decrease due to acid production from sugar consumption and an increase derived from the utilization of amino acids/proteins from the algae biomass being used as the sole nitrogen source.

### 2.4. Proposed Batch Valorization Strategies

**Figure 4:**
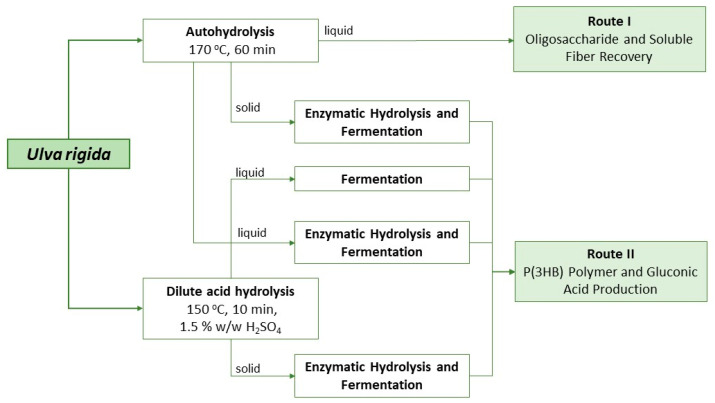
Schematic representation of different valorization strategies possible for the liquors produced from *Ulva rigida*.

From the results attained with shake flasks, possible valorization strategies for each liquor can be envisaged and are depicted in Figure 4.

Despite not being ideal for the large-scale production of polymer, the approach applied to liquor A can provide several functional valorization strategies for this biomass. After the autohydrolysis treatment, the liquor obtained contained not only simple sugars but also oligosaccharides (that were later converted into monosaccharides with enzymatic hydrolysis). Ulvan and its oligosaccharides are expected to behave as soluble fiber and to have potential prebiotic activity, as the common gastrointestinal enzymes are not able to break these marine saccharides’ bonds. Further, since liquor A does not seem to be the most promising for microbial cultivation purposes, the saccharification step is not needed and the direct use of the autohydrolysis liquor should be considered (Route I in Figure 4). At the operational conditions described, a total of 6 g/L of oligosaccharides was obtained, with half corresponding to xylose and rhamnose units. These types of compounds have several other reported bioactivities, such as immunomodulatory, anti-cancerous, anti-infection, anti-inflammatory and anti-lipidemic properties [27]. Moreover, this liquor is still rich in protein, which can improve its nutraceutical and functional properties. Furthermore, this valorization route is more environmentally and economically sustainable (due to the single-step approach with a lack of chemicals, resulting in shorter processing times and energy consumption) than the production of liquors to be used as substrates for microbial growth.

Route II is the strategy that aims at the total saccharification of *Ulva* polysaccharides to simple sugars to be used as substrates in microbial bioprocesses to produce, for instance, P(3HB) and/or gluconic acid. Due to the unavailability of a specific enzymatic cocktail targeting ulvan, saccharification of this complex polysaccharide must be carried out by a thermochemical treatment by autohydrolysis or dilute acid hydrolysis. Autohydrolysis occurs in milder conditions and thus both liquid and solid fractions need to undergo enzymatic treatment to produce monosaccharides. Commercially available enzymes such as cellulases and broad-spectrum carbohydrases such as CelicCtec2^®^ and Viscozyme^®^ are then used to target both cellulose (in the solid fraction) and ulvan (in the solid and liquid fractions), respectively. Alternatively, a dilute acidic treatment might be used but small amounts of furans are usually produced that need to be removed afterwards.

### 2.5. Fed-Batch Bioreactor Assays

With the results attained in shake flasks, fed-batch cultivations in a bench-scale stirred tank bioreactor (2 L) using *Ulva* liquors as feedstock were designed.

Due to their higher N content, Liquors A and C were considered adequate to support the growth phase, in particular Liquor C, as Liquor A exhibits a high viscosity that may hinder oxygen transfer in the bioreactor. Liquor C was thus chosen for the batch phase in the bioreactor. Liquor D was considered the most adequate to be used for polymer production for its low N content. As mentioned in Section 2.3, both liquors B and D were expected to show higher P(3HB) concentrations in the shake flask assays due to their similar sugar concentration and low N titers. However, a sharp drop in the pH values using Liquor B and the lack of pH control did not result in an effective polymer production. For this reason, liquor D was selected as a more adequate hydrolysate to target P(3HB) production and was chosen to be used as feed during the fed-batch phase. In fed-batch cultivations, a feed containing high sugar titers is necessary due to bioreactor volume constraints. This is possible through the concentration of large volumes of liquors produced after enzymatic hydrolysis of *Ulva* solids obtained from the acidic treatment. Due to the small volume of the available pressurized reactor (1 L), it was difficult to collect enough solids after the acidic treatment for subsequent enzymatic treatment. For this reason, *Ulva* residues obtained after the extraction of the protein fraction (and thus with a low N content), collected in the scope of a different project, were used to produce sugar-rich (and N-poor) hydrolysates for P(3HB) production after enzymatic hydrolysis of the cellulosic fraction. In fact, the protein fraction from the first extraction step of this biomass is also a source of functional ingredients. *Ulva*’s proteins are a reported source of essential amino acids which, alongside their prebiotic and anti-inflammatory potential and inhibitory capacity against the angiotensin I-converting enzyme, further increase the relevance of this approach.

**Figure 5:**
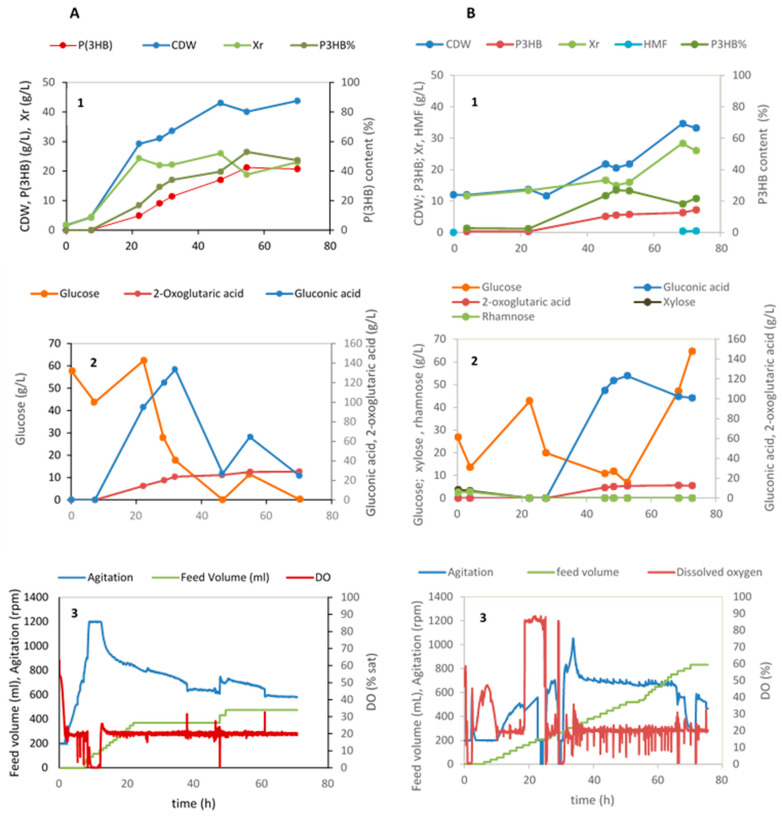
Fed-batch cultivation of *Halomonas elongata* 1H9^T^ under nitrogen-limiting conditions using commercial glucose (**A**) and *Ulva rigida* hydrolysates (**B**) as a carbon source (**1**) Cell biomass (Xr = CDW—P(3HB)), P(3HB) production and P(3HB) content (%), (**2**) sugar consumption and organic acids production; (**3**) data acquired automatically during the cultivation, namely, feed volume, agitation, and DO (% sat).

The results of the cultivation using these two hydrolysates are depicted in Figure 5B.

In the batch phase, the three sugars of the *Ulva* hydrolysate, i.e., glucose, xylose and rhamnose were consumed as expected. At approximately 6 h cultivation, pulses of the hydrolysate produced using the *Ulva* residues started being fed to maintain a glucose concentration in the medium of circa 20 g/L. In the period between 22 and 45 h, N-limited conditions were attained and P(3HB) started being produced. Also, during the same time interval a rise of the gluconic and 2-oxoglutaric acids was observed. These two metabolites produced by *H. elongata* have been identified in a previous study by the same authors [4], although the culture conditions that promote their synthesis are still not uncovered. Gluconic acid concentrations of circa 120 g/L have already been achieved by Leandro et al., 2023 [4] when using commercial glucose in the batch phase and concentrated glucose solutions as feed. The production of 2-oxoglutaric acid is now also given (Figure 5A,) attaining a final concentration of 29.1 g/L. 2-Oxoglutaric acid, also called alpha-ketoglutaric acid (KGA), is an intermediate of the Krebs cycle and of the amino acid metabolism, serving as starting point in the formation of glutamate and other members of the glutamate family, namely, glutamine, proline and arginine. Being the entry point for ammonia into the metabolism, glutamate is always important, but in the case of *H. elongata* it has a greater importance, since glutamate itself is an osmoregulatory solute and provides the amino groups for the synthesis of ectoine.

The assimilation of ammonia into amino acids is initiated by three biochemical reactions that form part of glutamate metabolism [28,29,30].

2-Ketoglutarate + NH_3_ + NAD(P)H ↔ glutamate + NAD(P)^+^ (1)

Glutamate + NH_3_ + ATP → glutamine + ADP + Pi (2)

Glutamine + 2-ketoglutarate + NAD(P)H → 2 glutamate + NAD(P)^+^ (3)

The first reaction is reversible and is catalyzed by glutamate dehydrogenases (GDHs). The sequential irreversible reactions (2) and (3) are catalyzed by glutamine synthetase (GS) and glutamate synthase (GOGAT), respectively [28]. In organisms that are able to accomplish the three reactions, ammonia will generally be assimilated via the GDH reaction when its extracellular concentration is high and via the GS/GOGAT route at low NH_3_ concentrations [28].

The production of 2-ketoglutarate (2-oxoglutarate) might be related to the depletion of the added glutamate in the initial medium.

KGA is of particular interest due to its broad application scope, namely, as a dietary supplement, a building block for the chemical synthesis of heterocycles, a constituent of wound healing compounds or as a main component of elastomers with a wide range of mechanical and chemical properties [31].

Some bacterial and yeast strains have been studied for their ability to produce KGA, as well as the conditions for its overproduction and secretion. Concerning *Halomonas* sp., only a previous work by our group using *H. boliviensis* [32] and a Japanese patent (JP2012170385A) [33] refer to the overproduction and excretion of this intermediate to the culture medium. The production of these two metabolites most probably competes for glucose and thus a lower yield of polymer on sugar was obtained. Assays to discern the culture conditions that improve the productivity of each of the two acids by *H. elongata* will be carried out. Like KGA, gluconic acid (and its derived gluconates) are also food additives used as acidity regulators and preservatives, as well as mineral supplements to treat hypocalcaemia, hypomagnesaemia and anaemia, possessing not only prebiotic but also antioxidant properties [34]. For these reasons, a valorization strategy centered around the production of these nutraceutical compounds should not be overlooked. Culture conditions that promote the production of these organic acids thus need to be further investigated, particularly the effect of oxygen concentration, glucose concentration and medium pH. High concentrations of oxygen and initial medium pH have been shown to significantly influence gluconic acid production. Likewise, conditions of low nitrogen and high glucose concentration favor gluconic acid production.

Concerning P(3HB), in the fed-batch cultivation with glucose, 21 g/L polymer was produced attaining a polymer content of 53% after 55 h cultivation corresponding to a volumetric productivity of 0.39 g P(3HB)/(L.h). When using hydrolysate B as the substrate for the batch phase, (Figure 5B), a lag phase of approximately 23 h takes place. In this phase, no cell growth or metabolite production is observed. This is probably related to the presence of furfural in the hydrolysate used in this phase (0.07 g/L; Table 2). An increase in growth and metabolite production (P(3HB), gluconic and 2-oxoglutaric acids) is observed after this point, although cell concentrations and titers of polymer obtained are much lower than the ones attained with commercial glucose. To calculate the maximum cell content of polymer attained, the solids present in the cultivation medium dragged by the hydrolysate were deduced from the total cell dry weight (CDW), considering that the total solids at the beginning of the cultivation are inert. A rough estimation of the maximum P(3HB) content can be made yielding a value of approx. 60%. This value is similar to the content of P(3HB) in the cultivation using glucose (53%, Figure 5A). The maximum polymer productivity attained in this assay was merely 0.11 g/(L.h). This low value was reached at 48.5 h cultivation. From then on, the rate of P(3HB) production lowered, although the available glucose in the medium kept increasing. Production levels of all three metabolites suffered an arrest after this moment, probably due to increasing concentrations of the inhibitor hydroxymethyl furfural (HMF) upon feed addition attaining a final value of 0.48 g/L (Figure 5B). The metabolism of *H. elongata* decreases with the increasing concentrations of this inhibitor. Previous assays have shown that its maximum specific growth rate (µ_max_ (h^−1^)) decreases 30% in the presence of 0.5 g/L HMF (results not published). The manufacture of *Ulva* hydrolysates aiming to achieve high sugar titers and no inhibitors is currently being improved.

## 3. Materials and Methods

### 3.1. Halomonas elongata 1H9^T^ Media Composition and Storage

*Halomonas elongata* 1H9^T^ (DSM 15516) was purchased from DSMZ-German Collection of Microorganisms and Cell Cultures GmbH. For storage, the strain was cultivated on a modified HM medium [4] and stored in cryovials at −80 °C with 15% (*w*/*v*) glycerol. Seed medium for inoculum preparation had the following composition: (per liter) 45 g NaCl, 2.5 g MgSO_4_·7H_2_O, 2.5 g NH_4_Cl, 0.55 g K_2_HPO_4_, 1 mL trace elements [35], 9 g monosodium glutamate, 15 g Tris-HCl, 20 g glucose. Nitrogen-limited medium for shake flask assays was composed of (per liter): 45 g NaCl, 2.5 g MgSO_4_·7H_2_O, 1 g NH_4_Cl, 3 g K_2_HPO_4_, 1 mL trace elements [35], 15 g Tris-HCl and 20 g sugar source. Both MgSO_4_·7H_2_O and sugars were supplemented to a sterile medium from concentrated solutions autoclaved separately.

### 3.2. Algae Biomass

*Ulva rigida* was obtained from AlgaPlus Lda, (Ílhavo, Portugal). The biomass was purchased dried and ground in flakes of 1.5–4 mm. Biomass was stored protected from light and humidity.

### 3.3. Ulva rigida Biomass Characterization

*Ulva rigida* samples were submitted to sequential exhaustive extractions with water (16 h) and ethanol:water 80:20 *w*/*w* (8 h) [36], followed by the analyses of total carbohydrates in the extracted fractions and residual biomass [37]. Moreover, the seaweed content in crude lipids (considered equal to ethanol extractives), protein (Kjeldahl [38], conversion factor of 5.13 [39]) and ash (calcination) [40] were determined.

### 3.4. Hydrolysis of the Carbohydrate Fraction of Whole Ulva rigida Biomass

*Ulva rigida* biomass was subjected to a hydrothermal hydrolysis (170 °C, 60 min, 10% solid loading) leading to the production of Liquor A which underwent a subsequent enzymatic treatment with Viscozyme^®^ L (Novozymes, Bagsværd, Denmark) (72 h, 50 °C, 180 rpm, 15 FPU/g, pH 5). This Liquor A was further treated with activated charcoal (120 min, stirring 1 g activated charcoal/10 g liquor) for removal of hydroxymethylfurfural (HMF). After the hydrothermal treatment, the remnant solid-phase was further subjected to enzymatic treatment with Viscozyme^®^ L and Cellic^®^ Ctec2 (Novozymes, Bagsværd, Denmark) (1:1) (72 h, 50 °C, 180 rpm, 15 FPU/g, pH 5) resulting in the production of Liquor B. Alternatively, *Ulva* rigida biomass was subjected to an acidic hydrolysis with 1.5% (*w*/*v*) sulfuric acid (150 °C, 10 min, 10% solid loading), resulting in the production of Liquor C which was further treated for removal of HMF with activated charcoal as described. The solids from the acidic treatment were further subjected to an enzymatic treatment with Viscozyme^®^ L and Cellic^®^ Ctec2 (1:1) (72 h, 50 °C, 180 rpm, 15 FPU/g, pH 5), resulting in the production of Liquor D. Both hydrothermal and acidic hydrolysis were performed in a 1.9 L 4520 Stirred Pressurized Bench Top Reactor (Parr Instruments Company, Moline, IL, USA). There was no external pressure applied to the reactor, i.e., it was determined by the vapor pressure of the liquid at each temperature. Nevertheless, it was monitored to guarantee that the extractions occurred in a subcritical state (being at 5 bar in the treatment performed at 150 °C and 8 bar in the treatment performed at 170 °C). Liquors A, B, C and D were concentrated up to around 20 g/L of total sugars in a VV2000 rotary evaporator (Heidolph Instruments, Schwabach, Germany).

### 3.5. Production of Concentrated Hydrolysates for the Bioreactor Assays

Liquor C was chosen as substrate for cell growth during the batch phase of the fed-batch cultivations. Aiming at this, the liquor was concentrated in a vacuum stove at 60 °C. A final sugar concentration of 76.6 g/L and HMF < 0.1 g/L was attained in the final concentrated hydrolysate.

Due to its low nitrogen content, Liquor D was considered the most appropriate hydrolysate to be used as sugar supply for P(3HB) production during the fed-batch phase of the cultivation. Instead of using whole algae to produce this hydrolysate, residues of *Ulva* obtained after protein extraction (in the scope of another project, results to be published) were used to produce glucose-rich, N- poor liquors. *Ulva rigida* residues were subjected to hydrothermal hydrolysis in an autoclave (121 °C, 30 min, 3.5% (*w*/*v*) solid loading), after which the supernatant was separated and the solids were subjected to enzymatic hydrolysis using a Cellic^®^ CTec2 (Novozymes, Bagsværd, Denmark) at 15 FPU/g, pH 5, 50 °C and 72 h to obtain glucose. After centrifugation and paper filtration to remove solids, the liquid was concentrated in a stove at 60 °C to a glucose concentration of 96 g/L. HMF was not detected.

### 3.6. Shake Flask Cultivations

Shake flask cultivations were performed to assess *H. elongata* 1H9^T^’s ability to grow and accumulate P(3HB) on the sugars present in *Ulva rigida* hydrolysates. The assays were carried out in 500 mL baffled flasks containing 100 mL of nitrogen-limited medium (see Section 3.1) designed to promote polymer synthesis upon nitrogen limitation. Sterile solutions of glucose (Dextropan 100- dextrose monohydrate COPAM, São João da Talha, Portugal), rhamnose (Carl Roth), xylose (D-xylose batch number 201904053, Shandong Lujian Biological, Shandong 251200—P.R. China) and glucuronic acid (PanReac AppliChem) were added to the culture medium following medium sterilization to attain a 20 g/L sugar concentration. Sugar consumption was tested separately in different assays.

To test *Ulva* hydrolysates prepared as described in Section 3.4 as a source of sugar and nitrogen, shake flask assays were also carried out. These assays aimed at the identification of the most adequate *Ulva*-derived sugar liquor for growth, polymer and gluconic acid production by *H. elongata* 1H9^T^. Assays were performed in 500 mL baffled flasks containing 50 mL of culture medium. All the components of N-limited medium (Section 3.1) were supplemented to the hydrolysate except the N-source, i.e., ammonium chloride. The nitrogen content found in the hydrolysates (N-total determined by the LECO method) was the sole source of N. As carbon source, 35 mL of *Ulva* rigida concentrated liquor (20 g/L total sugars) was used. From the literature, it is known that a C:N ratio of 20–40 favors PHA production [26]. To establish a C:N ratio of 20, commercial glucose was supplemented when needed. To avoid excessive dilution of the tested liquors, each medium component was supplemented from sterile concentrated stock solutions until a final volume of 50 mL. The initial medium pH was adjusted to pH 8. All shake flask cultures were incubated in an orbital shaker at 35 °C and 200 rpm (Agitorb 200, Aralab, Rio de Mouro Portugal).

Culture samples were harvested, and biomass, polymer and sugar concentrations followed.

### 3.7. Bioreactor Cultivations

Fed-batch cultivations were carried out in 2 L stirred-tank reactors (Bioflo115, New Brunswick Scientific, Edison, NJ, USA) with an initial working volume of 1.3 L. The bioreactors are equipped with 2 six-bladed disk-turbine impellers and are operated with the software BioCommand Batch Control, which enables control, monitoring and data acquisition. An inoculum of 130 mL (10% (v_inoc_/v_bioreactor_)) was previously prepared in 500 L flasks with seed medium (Section 3.1) and inoculated with two cryovials. Flasks were placed in an orbital incubator (Infors AG, Bottmingen, Switzerland) at 35 °C, 200 rpm for 18 h attaining a cell density (OD600) in the range 5–8. After bioreactor inoculation, temperature and pH were maintained at 35 °C and 7.5, respectively. For pH control, NaOH (5–10 M) was used. Dissolved oxygen concentration was maintained at 20% saturation by controlling the stirring speed between 200 and 1200 rpm in cascade mode with the dissolved oxygen and using a constant aeration rate of 2.6 Lair/min. Foam was detected and controlled through the intermittent addition of Simethicone Emulsion USP, Dow Corning as an anti-foam agent.

The initial bioreactor medium composition was (1 L): 45 g NaCl; 5 g MgSO_4_·7H_2_O; 12 g NH_4_Cl; 6 g K_2_HPO_4_; 7.7 mL Trace elements SL-10 solution [35], 10 g monosodium glutamate monohydrate (MSG) and 50 g glucose. Glucose, MgSO_4_·7H_2_O and MSG concentrated solutions (500 g/L, 100 g/L and 250 g/L, respectively) were previously sterilized and added aseptically to the other medium components that had been sterilized inside the bioreactor. Glucose and magnesium ion solutions were autoclaved, and MSG concentrated solution (250 g/L) was filter-sterilized.

Cultivations were designed to endorse cell growth during the batch phase up to 20–25 g/L cell concentration (CDW). Thereafter and upon nitrogen limitation, P(3HB) production was promoted. This took place during the fed-batch stage along which the feed solution was added by pre-programmed feeding pulses using the controlling software. The feed composition was 600 g/L glucose, 5 g/L MgSO_4_·7H_2_O and 45 g/L NaCl.

When *Ulva* hydrolysates were used as C-source, 800 mL of concentrated liquor C was used to attain an initial sugar concentration of 50 g/L in the batch phase. Liquor C was autoclaved inside the bioreactor and, after cooling, concentrated sterile solutions of all the other components were added. The volume of a concentrated NH_4_Cl solution added to the hydrolysate took into consideration the nitrogen titer of this liquor (9.4 g/L N). In the fed-batch phase, 422.5 mL of the hydrolysate produced with *Ulva* residues supplemented with glucose to attain a glucose concentration of 410 g/L and NaCl to a concentration of 45 g/L was used as feed. The composition of the hydrolysates and that of the final feed is given in Table 2.

Culture samples were periodically harvested and analyzed for biomass, polymer and sugar concentrations.

### 3.8. Analytical Methods

Cellular growth was monitored off-line by measuring the optical density (OD) of culture samples at 600 nm with a double beam spectrophotometer (Hitachi U-2000, Hitachi High-Tech Science Corporation, Tokyo, Japan). For cell dry weight analysis, 1.2 mL culture samples were centrifuged in a Sigma 1–15 P microcentrifuge (9168× *g*, 5 min) in pre-weighed and dried microtubes. Cell pellets were then washed with distilled water, centrifuged for water removal and dried at 62 °C in an oven (Model 400, Memmert GmbH, Schwabach, Germany) until constant weight.

Sugars such as glucose, xylose and rhamnose, organic acids such as glucuronic, gluconic and 2-oxoglutaric acids, and the furans furfural and hydroxymethylfurfural concentrations were determined using an HPLC (Hitachi LaChrom Elite, Hitachi High-Tech Science Corporation, Tokyo, Japan) equipped with a Rezex ROA-Organic acid H^+^ 8% (300 mm × 7.8 mm) column, an autosampler (Hitachi LaChrom Elite L-2200), a HPLC pump (Hitachi LaChrom Elite L-2130), a Hitachi L-2490 refraction index detector (RI) and a Hitachi L-2420 UV-Vis detector. Sugars were detected by the RI while the detection of organic acids, furfural and HMF was carried out by the UV detector. A column heater for larger columns (Croco-CIL 100-040-220P SCP Seitz Chromatographie Produkte GmbH, Weiterstad, Germany, 40 cm × 8 cm × 8 cm, 30–99 °C) was connected externally to the HPLC system. The injection volume was 20 μL and elution was achieved using a 5 mM solution of H_2_SO_4_. The column was kept at 65 °C, and the pump operated at a flow rate of 0.5 mL/min. Solutions of glucose (Merck), gluconic acid (Sigma-Aldrich), rhamnose (Carl Roth), xylose (Sigma-Aldrich), glucuronic acid (PanReac AppliChem) and hydroxymethylfurfural (Carbosynth) were used to prepare standard curves for each compound for posterior peak detection and quantification in culture samples. Oligosaccharide content was calculated based on the increase in monosaccharide content after a quantitative post-hydrolysis (121 °C, 20 min, 4% H_2_SO_4_), versus a direct analysis of the liquor [41]. For P(3HB) quantification, 1.2 mL culture samples were taken and centrifuged in a Sigma 1–15 P microcentrifuge (9168× *g*, 5 min) to recover cell pellets which were subjected to acidic methanolysis for PHA extraction as described before [23].

Total nitrogen in the hydrolysates was quantified by an analyzer FP-528 DSP LECO (LECO, St. Joseph, MI, USA) which uses the Dumas method for N quantification [42].

### 3.9. Statistical Analysis

Chemical composition of *Ulva rigida* biomass and production and characterization of liquors was conducted using at least three replicates (*n* ≥ 3). When possible, values are reported as averages with standard deviation.

## 4. Conclusions

An integrated, sequential valorization of the green seaweed *Ulva rigida* was proposed after optimization of the hydrolytic process for saccharification of the polysaccharide fraction of this alga. Depending on the treatment, the produced liquors are rich in oligosaccharides or in simple sugars such as glucose, with minor concentrations of xylose and rhamnose and uronic acids such as glucuronic acid. These sugars were used for the co-production of P(3HB) and gluconic acid by *H. elongata* 1H9^T^. Based on preliminary studies in shake flasks, fed-batch cultivations at bench-scale were designed. The liquid fraction after the acidic treatment resulted in a N-rich liquor suited to promote growth, while the enzymatic treatment of the derived solid fraction resulted in a N-poor liquor appropriate to be used as feed in the second stage of fed-batch cultivations for P(3HB) production. Fed-batch cultivations using the obtained liquors under nitrogen-limiting conditions produced P(3HB) as well as high titers of gluconic acid while 2-oxoglutaric acid was also produced at lower concentrations. These organic acids have multiple applications including as dietary supplements and their production potentially contributes to increasing the economic competitiveness of bioplastic production. Alternatively, hydrothermal treatments can create liquors with ulvan oligosaccharides with important bioactive and functional properties. Hence, this work broadens the options to process one of the most widespread marine macroalgae and find the best routes for its valorization.

## Figures and Tables

**Table 1:** Chemical composition of *Ulva rigida* biomass.

Component	g/100 g Dry Weight
Ash	33.32 ± 0.22
Protein	11.35 ± 0.36
Lipids (ethanol extractives)	2.56 ± 0.26
Water extractives	40.22 ± 1.88
Acid Insoluble Residues	12.40 ± 0.31
Glucuronic acid	1.28 ± 0.03
Glucan	11.87 ± 0.89
Xylan/Galactan/Mannan	10.34 ± 1.42
Arabinan	1.98 ± 0.24
Rhamnan	8.95 ± 0.17

**Table 2:** Hydrolysates and final feed composition in the fed-batch cultivations.

	*Ulva* Hydrolysate Used in the Batch Phase (g/L)	*Ulva* Hydrolysate Used in the Fed-Batch Phase (g/L)	Supplemented *Ulva* Hydrolysate Used as Feed in the Fed-Batch Phase (g/L)
Glucose	19	117.8	410
Xylose	15	12.5	6.2
Rhamnose	10	-	-
HMF	0.05	0.97	0.48
Furfural	0.07	-	-

## Data Availability

Not applicable.
